# Effect of Vitamin D on Blood Pressure and Hypertension in the General Population: An Update Meta-Analysis of Cohort Studies and Randomized Controlled Trials

**DOI:** 10.5888/pcd17.190307

**Published:** 2020-01-09

**Authors:** Dongdong Zhang, Cheng Cheng, Yan Wang, Hualei Sun, Songcheng Yu, Yuan Xue, Yiming Liu, Wenjie Li, Xing Li

**Affiliations:** 1 Department of Nutrition and Food Hygiene, College of Public Health, Zhengzhou University, Henan, China; 2 Department of Epidemiology and Health Statistics, College of Public Health, Zhengzhou University, Henan, China

## Abstract

**Background:**

The effect of vitamin D supplementation on blood pressure has been explored in previous meta-analyses, but whether the association is causal in the general population is still unknown. We evaluated the association comprehensively and quantitatively.

**Methods:**

We searched PubMed and Embase for relevant cohort studies and randomized controlled trials (RCTs). We used a 2-step generalized least-squares method to assess the dose–response association of circulating 25-hydroxyvitamin D (25[OH]D) and hypertension and a fixed-effects model to pool the weighted mean differences (WMDs) and corresponding 95% confidence intervals (95% CIs) of blood pressure across RCTs.

**Results:**

We identified 11 cohort studies and 27 RCTs, with 43,320 and 3,810 participants, respectively. The dose–response relationship between circulating 25(OH)D levels and hypertension risk was approximately L-shaped (*P*_nonlinearity_ = .04), suggesting that the risk of hypertension increased substantially below 75 nmol/L as 25(OH)D decreased, but it remained significant over the range of 75–130 nmol/L. However, pooled results of RCTs showed that there was no significant reduction in systolic blood pressure (WMD, −0.00 mm Hg; 95% CI, −0.71 to 0.71) or diastolic blood pressure (WMD, 0.19 mm Hg; 95% CI, −0.29 to 0.67) after vitamin D intervention.

**Conclusions:**

The results of this meta-analysis indicate that supplementation with vitamin D does not lower blood pressure in the general population. RCTs with long-term interventions and a sufficient number of participants who have low levels of vitamin D are needed to validate these findings.

**Summary**

**What is already known on this topic?**

The effects of vitamin D on hypertension risk and blood pressure have been explored widely in cohort studies and randomized controlled trials (RCTs), but whether the association is causal still is unknown.

**What is added by this report?**

We performed an update meta-analysis of both cohort studies and RCTs in a generally heathy population and found that the dose–response relationship between circulating 25-hydroxyvitamin D level and hypertension risk was approximately L-shaped. However, pooled results of RCTs showed that there was still no significant reduction in systolic and diastolic blood pressure.

**What are the implications for public health practice?**

Vitamin D supplementation is ineffective to prevent hypertension.

## Introduction

Emerging evidence suggests that vitamin D deficiency is a widespread global problem (1). According to the Institute of Medicine (IOM), vitamin D deficiency is defined as circulating 25-hydroxyvitamin D (25[OH]D) level <50 nmol/L based on the optimal concentration for skeletal health (2). Interest has increased concerning the potential health consequences of vitamin D deficiency, such as increased risk of cardiovascular diseases, cancers, and Alzheimer’s disease (3–5). Although observational data have demonstrated that poor vitamin D status is associated with increased risk of hypertension (6–9), randomized controlled trials (RCTs) have provided little support for the beneficial effect of vitamin D supplementation on blood pressure (10–13). Considering the potential residual confounding, inferring causality or reversibility of this relationship and reaching consensus from these findings is difficult.

Several meta-analyses of observational studies and RCTs have been published, but results are conflicting (14–17). Golzarand et al evaluated 30 RCTs with 4,744 participants and concluded that vitamin D has a beneficial effect in subgroups of daily doses >800 IU/d, a duration less than 6 months, or older subjects (14). Kunutsor et al suggested that supplementation with vitamin D significantly reduced diastolic blood pressure (DBP) by 1.31 mm Hg in participants with preexisting cardiometabolic conditions (16). However, another meta-analysis performed by incorporating individual data supported that vitamin D supplementation is ineffective in lowering blood pressure (15).

Taken together, it may be hypothesized that the increased blood pressure or risk of hypertension is partly explained by individuals’ baseline vitamin D status, the sample size, the intervention dose, and the follow-up duration. Meanwhile, considering that pre-existing conditions such as diabetes, cardiovascular disease, and kidney disease may influence the physiologic mechanism of vitamin D on blood pressure, considerable variability may exist between individual patients and the general population. Therefore, restricting the participants to the general population may help to explore the true association hidden by the confounders. Analyzing the population as a whole rather than restricting analyses to certain population subgroups may help us to explore the true association hidden by confounders. In addition, results from at least 10 more studies including 1,716 participants have been published on this topic since the latest meta-analysis in 2015 (10–12,18–24).

We aimed to provide a comprehensive and quantitative meta-analysis from the published cohort studies and RCTs on the effect of vitamin D involving hypertension risk and blood pressure levels in the general population.

## Methods

We used the PRISMA (Preferred Reporting Items for Systematic Review and Meta-Analyses) checklist to perform the meta-analysis and report the results (25).

### Data source and searches

We searched PubMed and Embase databases up to June 12, 2019, for cohort studies reporting an association between blood 25(OH)D levels and risk of incident hypertension and for RCTs examining the effect of vitamin D supplementation (alone or in combination with other nutrients) on blood pressure. The search terms “vitamin D” and “blood pressure” were used in combination to retrieve relevant records. The records were restricted to human studies, and additional studies were retrieved through manually searching the references of identified articles and relevant systematic reviews. 

### Study selection

Two investigators (D.Z. and C.C.) reviewed the titles and abstracts independently to identify articles for potentially relevant sources. Full-text versions were requested to evaluate eligibility. To be included, the study had to meet the following criteria: 1) followed an RCT or a cohort study design; 2) investigated the association between vitamin D and risk of hypertension or effect of blood pressure levels; 3) included a general population (≥18 y) rather than patients with specific diseases (eg, diabetes, hypertension, stroke, heart failure); and 4) provided estimates of the risks of hypertension in at least 3 categories of blood 25(OH)D levels or reported continuous risk estimates for the dose–response analysis, or reported blood pressure for meta-analysis of RCTs. We excluded articles if they 1) measured other metabolites of vitamin D (eg, 1,25-dihydroxyvitamin D); 2) focused on pregnant women or groups with specific diseases; or 3) did not report blood pressure at baseline/end or the changes after invention from baseline for trials. Inconsistencies were resolved through group discussion or adjudicated by a third reviewer.

### Data extraction

Using predefined protocols, D.Z. extracted data from each study and C.C. checked the accuracy. For cohort studies, the following information was abstracted: first author, publication year, country, follow-up period, sample size, age, number of cases/participants, categories of 25(OH)D levels, reported risk estimates, 95% confidence intervals (CIs), and covariates adjusted for in the analyses. When several adjusted models were explored, we extracted the risk ratios from the model with largest number of covariables. If the lowest 25(OH)D level was not the reference, we recalculated the risk estimates by the method of Hamling et al (26). When the mean or median 25(OH)D level per category was not reported, we assigned the value as the midpoint of the lower and upper bound in each category (27). If the category was open-ended, we assumed the width of interval to be the same as in the adjacent category (27). If studies reported 25(OH)D levels in ng/mL, we converted the values to nmol/L by multiplying by 2.5.

For RCTs, we recorded the following data: study design (sample size of each group, blinding methods, intervention/placebo type and amount, duration of intervention, type of vitamin D, and intervention frequency); characteristics of participants (age, sex, baseline circulating 25[OH]D levels); and baseline/end blood pressure in both intervention and placebo groups and/or blood pressure changes from baseline. If studies used different doses of vitamin D, we extracted only the highest dose in the analysis. If studies measured blood pressures repetitively at different intervals during the intervention, we included only the blood pressure values at the longest follow-up point. Attempts were made to contact corresponding authors for unavailable information.

### Risk for bias assessment

We used the 9-star Newcastle–Ottawa Scale to evaluate the quality of individual cohort studies; the scale is based on 8 aspects covering selection, comparability, and outcome domains (28). Meanwhile, we assessed the risk of bias for each trial using 7 fields from The Cochrane Collaboration’s tool: random sequence generation, allocation concealment, blinding of participants and personnel, blinding of outcome assessment, incomplete outcome data, selective reporting, and other bias (29). Summary assessments for trials were assigned as “high,” “low,” or “unclear,” according to the risk bias in each outcome. Disagreements were resolved through group discussion. Publication bias was assessed with Egger’s test (30).

### Data synthesis and analysis

To provide dose–response evidence from all cohort studies, we used the 2-step generalized least-squares method (31). Study-specific slope coefficients were examined by restricted cubic splines with three knots at 25%, 50%, and 75% of the distribution of circulating 25(OH)D levels. For the dose–response analyses of 25(OH)D, the reference category was re-scaled to 75 nmol/L, which is the cutoff value between insufficient and sufficient vitamin D status. *P* values for nonlinearity were calculated by using the Wald χ^2^ test, assuming the coefficient of the second spline was zero. We used the DerSimonian and Laird random effects model to estimate the study-specific dose–response risk, and we calculated the pooled risk of hypertension for every 25 nmol/L increment in 25(OH)D levels using a random effects model (32).

We assessed the effect of vitamin D supplementation by the mean blood pressure changes (including systolic blood pressure [SBP] and DBP) in the intervention group minus the changes in blood pressure in the placebo group. The standard deviations (SDs) were obtained as reported or calculated from 95% CIs, *P* values for *t* statistics, or individual standard errors (SE) from intervention and placebo groups. If the studies did not report blood pressure changes from baseline, we calculated the mean values by using blood pressure after intervention minus blood pressure at baseline, and the SD of changes was obtained according the following formula, described in the *Cochrane Handbook for Systematic Reviews of Interventions* (29):

${SD}_{change}=\sqrt{{SD}_{baseline}^{2}+{SD}_{final}^{2}-(2\times Corr\times {SD}_{baseline}\times {SD}_{final})}$

We estimated correlation by calculations from 2 studies that provided complete data for SD_baseline_, SD_final_, SD_change_ in both intervention and placebo groups (33,34). Between-study heterogeneity was assessed with the *I* ^2^ and Q statistics. We used fixed-effects models and forest plots to pool the weighted mean differences (WMDs) and corresponding 95% CIs of blood pressure across studies.

Predefined subgroup analyses were performed to explore potential effect modification and sources of heterogeneity. We also conducted sensitivity analyses by removing one study at a time to ensure that the pooled result was not simply dependent on one large or individual case. All statistics were analyzed using Stata, version 12.1 (StataCorp, LLC). Significance was set at *P* < .05.

## Results

### Descriptive study characteristics

The systematic search in PubMed and Embase retrieved 8,956 publications, and 3 more were identified by manual searching. After duplicate checking and initial review of the titles and abstracts, 156 potentially relevant articles were obtained in full text for further evaluation. Finally, 119 articles were excluded and 37 publications (including 11 cohort studies in 10 publications [6–9,35–40] and 27 trials [10–13,18–24,33,34,41–54]) were eligible for inclusion.

Eleven cohort studies with 8,397 incident cases of hypertension and 43,320 participants were identified from 10 publications. With the exception of 1 study conducted in Asia, most were conducted in Europe (n = 4) and the United States (n = 6). The follow-up durations ranged from 1.3 to 15.3 years (median 5.0 years). Analyses of the quality of studies yielded an average NOS score of 7.5, nine of which were of high quality (score ≥7).

Twenty-seven studies were RCTs with 3,810 participants. Among them, 2 studies included only men, 10 included only women, and 15 included both. Five of the included trials were conducted in Asia, 12 were performed in Europe, 4 were conducted in Oceania, and the remaining 6 were performed in the United States. Mean or median baseline 25(OH)D concentrations varied from 25.6 nmol/L to 78.0 nmol/L, and 11 studies investigated the effects in individuals with vitamin D insufficiency, vitamin D deficiency, or both. Nine trials did not provide the final 25(OH)D concentration in intervention arms, whereas the remaining studies showed a substantial increase in circulating levels of 25(OH)D compared with the baseline assessment. All trials had low risk of bias for random allocation and selective reporting. There was insufficient information about allocation concealment in 5 trials and high risk of bias in 1 trial. One open-label trial had high risk of bias for blinding of participants and personnel and unclear bias risk for blinding of outcome assessment (43).

### Meta-analyses results

#### Circulating 25(OH)D levels and hypertension risk

**Figure 1:**
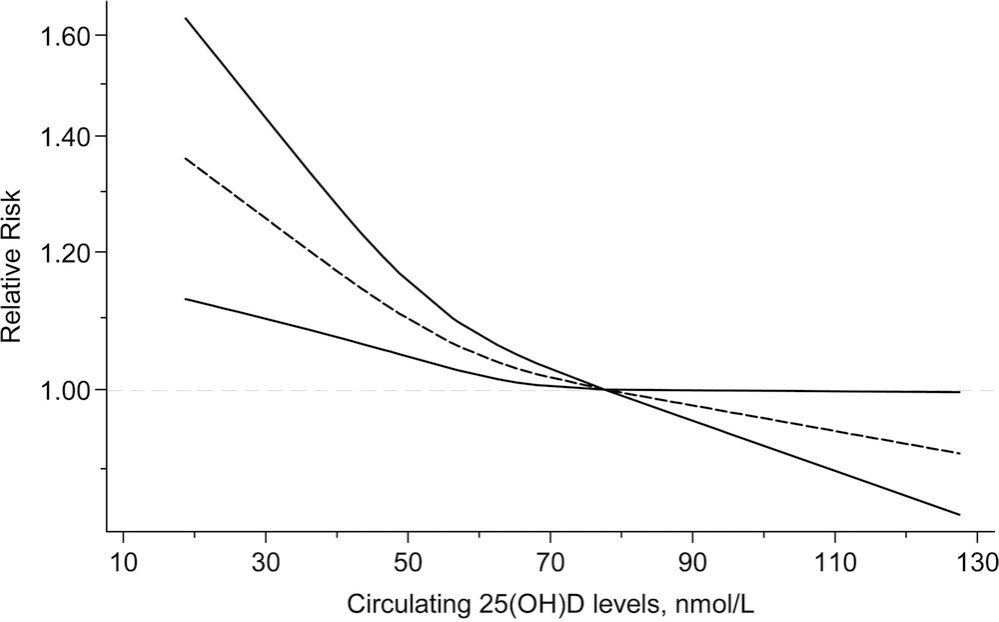
Nonlinear dose–response association between circulating 25(OH)D levels and hypertension risk, update meta-analysis of cohort studies of the effect of 25(OH)D levels on hypertension in the general population. The dashed line indicates the pooled restricted cubic spline model, and the solid lines indicate the 95% CIs of the pooled curve. Abbreviations: 25(OH)D, 25-hydroxyvitamin D; CI, confidence interval.

Quantitative results from meta-analyses of cohort studies showed that the risk of incident hypertension decreased by 7% (relative risk [RR] = 0.93; 95% CI, 0.89–0.98) per 25 nmol/L increment in 25(OH)D levels, with significant heterogeneity (*I* ^2^ = 61.6%, *P*_heterogeneity_ = .004). Ten studies reporting RR for 25(OH)D exposures in at least 3 levels were eligible for the linear trend estimation. Results from the analysis of restricted cubic splines indicated an approximate L-shaped correlation between circulating 25(OH)D levels and hypertension risk (*P*_nonlinearity_ = .04, Figure 1). The risk of hypertension increased substantially below 75 nmol/L as 25(OH)D decreased but remained significant over the range of 75–130 nmol/L.

**Table:** 

Circulating 25(OH)D levels, nmol/L	Relative risk (95% CI)
30	1.26 (1.10–1.43)
50	1.10 (1.01–1.16)
75	1 [Reference]
90	0.98 (0.96–1.00)
110	0.94 (0.89–1.00)

Subgroup analyses indicated sex (male, female, or mixed), follow-up duration (≤5 y or >5 y), region (America, Europe, or Asia), number of cases (<1,000 or ≥1,000), and study quality (high, medium, or low) as the potential sources of the heterogeneity (Table 1). However, the association of 25(OH)D levels per 25 nmol/L increment showed no significance in subgroups of men (RR = 0.93; 95% CI, 0.85–1.00), women (RR = 0.88; 95% CI, 0.76–1.01), European region (RR = 0.97; 95% CI, 0.94–1.01), small number of cases (RR = 0.95; 95% CI, 0.89–1.02), and medium or low quality of study (RR = 0.91; 95% CI, 0.80–1.03). Furthermore, the pooled estimates could not be altered substantially by removing one study at a time, and we found no evidence of publication bias by Egger’s test (*P* = .38).

**Table 1:** Subgroup Analyses for the Dose–Response Association Between Per 25 nmol/L Increment in Circulating 25-Hydroxyvitamin D and Hypertension Risk, Update Meta-Analysis of Cohort Studies, 2019

Subgroup	No. of studies	No. of participants	RR (95% CI)	*P* value	*I* ^2^, %
**Sex**					
Male	3	3,230	0.93 (0.85–1.00)	.06	28.7
Female	2	3,351	0.88 (0.76–1.01)	.07	0
Mixed	6	36,739	0.95 (0.89–1.00)	.06	76.4
**Region**					
United States	6	30,002	0.90 (0.83–0.97)	.006	65.1
Europe	4	10,862	0.97 (0.94–1.01)	.11	0
Asia	1	2,456	0.97 (0.90–1.05)	.44	—
**No. of cases**					
<1,000	6	5,696	0.95 (0.89–1.02)	.16	39.6
≥1,000	5	37,624	0.94 (0.91–0.96)	.02	77.1
**Duration, years**					
≤5	6	31,171	0.92 (0.84–1.00)	.06	73.9
>5	5	12,149	0.96 (0.93–0.99)	.01	0
**Study quality**					
High	7	18,488	0.96 (0.94–0.99)	.006	9.5
Medium or low	2	24,832	0.91 (0.80–1.03)	.13	87.0

Abbreviations: — , not applicable/not calculated; CI, confidence interval; RR, relative risk.

### Vitamin D supplementation and blood pressure levels

**Figure 2:**
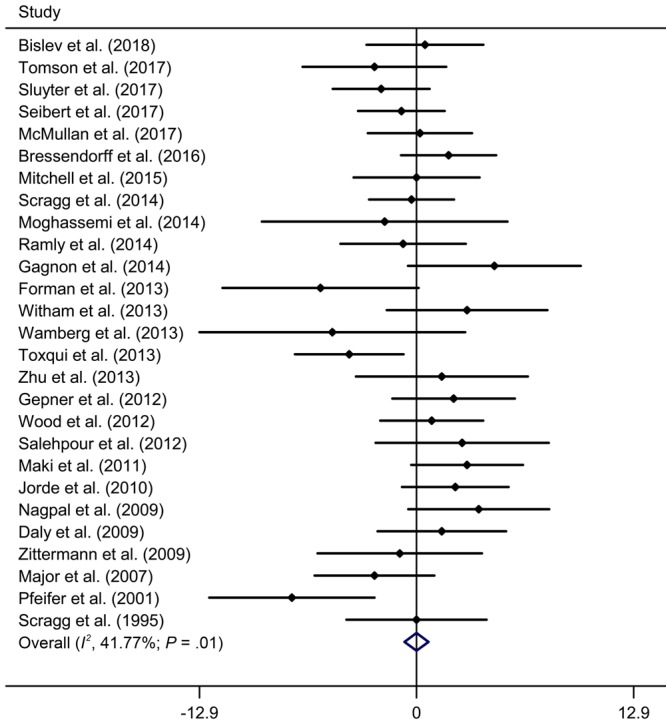
Meta-analysis of effect of vitamin D supplementation on systolic blood pressure, update meta-analysis of randomized controlled trials of the effect of vitamin D on blood pressure in the general population. Abbreviations: CI, confidence interval; WMD, weighted mean difference.

**Figure 3:**
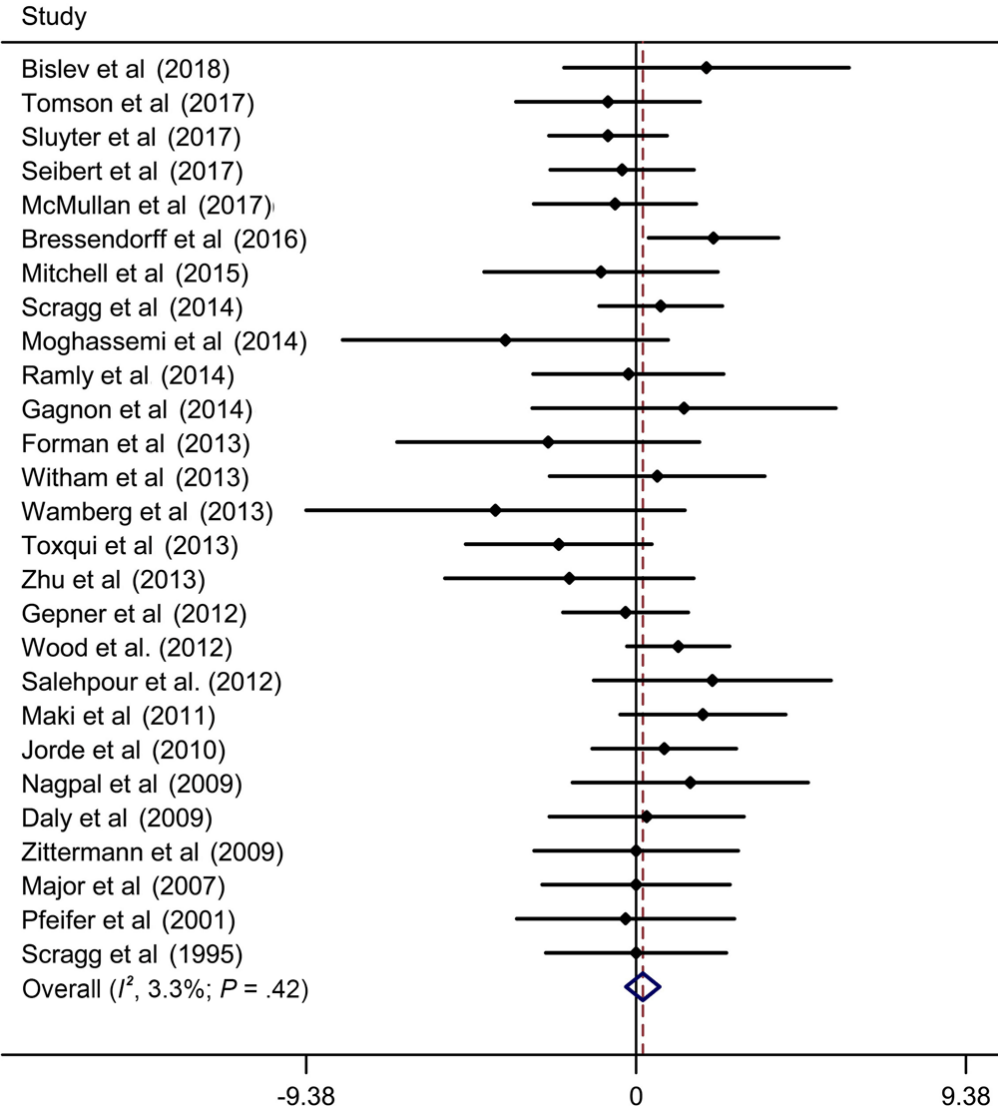
Meta-analysis of effect of vitamin D supplementation on diastolic blood pressure, update meta-analysis of randomized controlled trials of the effect of vitamin D on blood pressure in the general population. Abbreviation: WMD, weighted mean difference.

Figures 2 and 3 present the forest plots for effect of vitamin D supplementation on SBP and DPB across the included 27 trials. Overall, vitamin D supplementation did not have a significant effect on SBP reduction (WMD, −0.00 mm Hg; 95% CI, −0.71 to 0.71), with evidence of low heterogeneity (*I* ^2^ = 41.7%, *P*_heterogeneity_ = .01). There was also no significant reduction in DBP after intervention, and the WMD (95% CI) was 0.19 mm Hg (−0.29 to 0.67), without evidence of significant heterogeneity (*I* ^2^ = 3.3%, *P*_heterogeneity_ = .42).

**Table:** 

Study (year)	WMD, systolic blood pressure, mm Hg (95% CI)	Weighted %
Bislev et al (2018)	0.50 (−2.97 to 3.97)	4.20
Tomson et al (2017)	−2.50 (−6.76 to 1.76)	2.78
Sluyter et al (2017)	−2.10 (−4.98 to 0.78)	6.11
Seibert et al (2017)	−0.90 (−3.48 to 1.68)	7.62
McMulllan et al (2017)	0.20 (−2.89 to 3.29)	5.28
Bressendorff et al (2016)	1.90 (−0.93 to 4.73)	6.33
Mitchell et al (2015)	0 (−3.74 to 3.74)	3.61
Scragg et al (2014)	−0.30 (−2.82 to 2.22)	7.93
Moghassemi et al (2014)	−1.89 (−9.18 to 5.40)	0.95
Ramly et al (2014)	−0.80 (−4.52 to 2.92)	3.65
Gagnon et al (2014)	4.62 (−0.51 to 9.75)	1.92
Forman et al (2013)	−5.70 (−11.52 to 0.12)	1.49
Witham et al (2013)	3.00 (−1.77 to 7.77)	2.23
Wamberg et al (2013)	−5.00 (−12.89 to 2.89)	0.81
Toxqui et al (2013)	−4.00 (−7.23 to −0.77)	4.86
Zhu et al (2013)	1.50 (−3.60 to 6.60)	1.94
Gepner et al (2012)	2.20 (−1.44 to 5.84)	3.82
Wood et al (2012)	0.90 (−2.16 to 3.96)	5.41
Salehpour et al (2012)	2.71 (−2.43 to 7.85)	1.91
Maki et al (2011)	3.00 (−0.32 to 6.32)	4.58
Jorde et al (2010)	2.30 (−0.86 to 5.46)	5.06
Nagpal et al (2009)	3.69 (−0.49 to 7.87)	2.89
Daly et al (2009)	1.50 (−2.31 to 5.31)	3.47
Zittermann et al (2009)	−1.00 (−5.88 to 3.88)	2.12
Major et al (2007)	−2.50 (−6.06 to 1.06)	4.00
Pfeifer et al (2001)	−7.40 (−12.31 to −2.49)	2.10
Scragg et al (1995)	0 (−4.16 to 4.16)	2.92
Overall (*I* ^2^, 41.7%; *P* = .01)	0 (−0.71 to 0.71)	100.0

**Table:** 

Study (year)	WMD, diastolic blood pressure, mm Hg (95% CI)	Weighted %
Bislev et al (2018)	2.00 (−2.05 to 6.05)	1.39
Tomson et al (2017)	−0.80 (−3.42 to 1.82)	3.32
Sluyter et al (2017)	−0.80 (−2.48 to 0.88)	8.13
Seibert et al (2017)	−0.40 (−2.44 to 1.64)	5.48
McMulllan et al (2017)	−0.60 (−2.91 to 1.71)	4.28
Bressendorff et al (2016)	2.20 (0.35 to 4.05)	6.68
Mitchell et al (2015)	−1.00 (−4.33 to 2.33)	2.06
Scragg et al (2014)	0.70 (−1.05 to 2.45)	7.43
Moghassemi et al (2014)	−3.72 (−8.35 to 0.91)	1.07
Ramly et al (2014)	−0.22 (−2.93 to 2.49)	3.11
Gagnon et al (2014)	1.36 (−2.96 to 5.68)	1.22
Forman et al (2013)	−2.50 (−6.80 to 1.80)	1.24
Witham et al (2013)	0.60 (−2.46 to 3.66)	2.44
Wamberg et al (2013)	−4.00 (−9.38 to 1.38)	0.79
Toxqui et al (2013)	−2.20 (−4.84 to 0.44)	3.27
Zhu et al (2013)	−1.90 (−5.44 to 1.64)	1.82
Gepner et al (2012)	−0.30 (−2.08 to 1.48)	7.20
Wood et al (2012)	1.20 (−0.26 to 2.66)	10.73
Salehpour et al (2012)	2.17 (−1.20 to 5.54)	2.00
Maki et al (2011)	1.90 (−0.45 to 4.25)	4.12
Jorde et al (2010)	0.80 (−1.25 to 2.85)	5.42
Nagpal et al (2009)	1.54 (−1.81 to 4.89)	2.03
Daly et al (2009)	0.30 (−2.46 to 3.06)	2.99
Zittermann et al (2009)	0 (−2.90 to 2.90)	2.71
Major et al (2007)	0 (−2.67 to 2.67)	3.20
Pfeifer et al (2001)	−0.30 (−3.40 to 2.80)	2.38
Scragg et al (1995)	0 (−2.57 to 2.57)	3.47
Overall (*I* ^2^, 3.3%; *P* = .42)	0.19 (−0.29 to 0.67)	100.0

Table 2 shows the subgroup analyses of summary WMDs in SBP and DBP. We found that the heterogeneity decreased in studies of men, studies with overweight or obese individuals, studies with a large sample size (≥200), and studies with an intervention duration of 6 months or longer. The effects of vitamin D supplementation on SBP and DBP was still insignificant in all subgroups. In sensitivity analyses, the summary results remained similar by removing one study at a time. According to Egger’s test, we found no evidence of publication bias in studies of SBP (*P* = .60) and DBP (*P* = .07).

**Table 2:** Subgroup Analyses of Vitamin D Supplementation and Blood Pressure Levels in the General Population, Update Meta-Analysis of Randomized Controlled Trials, 2019

Subgroup	No. of Studies	No. of Participants	SBP			DBP		
			WMD (95% CI)	*P*	*I* ^2^, %	WMD (95% CI)	*P*	*I* ^2^, %
**Sex**								
Male	2	211	2.49 (−0.33 to 5.31)	.08	0	0.80 (−1.33 to 2.93)	.46	0
Female	10	1,215	−0.68 (−2.59 to 1.23)	.48	55.5	0.18 (−0.60 to 0.97)	.65	13.2
Mixed	15	2,384	0.11 (−0.81 to 1.02)	.82	28.6	0.14 (−0.49 to 0.76)	.66	11.7
**Age, y**								
<50	15	1,751	0.04 (−0.88 to 0.96)	.93	29.7	0.23 (−0.43 to 0.88)	.50	9.1
≥50	12	2,059	−0.27 (−2.01 to 1.48)	.76	55.5	0.15 (−0.55 to 0.84)	.68	4.0
**Region**								
United States	6	569	−0.01 (−2.17 to 2.14)	.99	50.3	−0.09 (−1.11 to 0.92)	.86	0
Europe	12	1,698	−0.61 (−2.20 to 0.97)	.45	52.9	0.42 (−0.27 to 1.11)	.23	19.1
Asia	5	469	1.24 (−0.87 to 3.35)	.25	0	−0.06 (−1.57 to 1.44)	.94	33.4
Oceania	4	1,074	−0.06 (−1.67 to 1.56)	.94	48.4	0.06 (−1.01 to 1.14)	.91	0
**Baseline obesity status**								
Overweight/obese	9	895	1.01 (−0.32 to 2.34)	.14	26.9	0.40 (−0.53 to 1.33)	.40	3.4
Not clear^a^	18	2,915	−0.41 (−1.25 to 0.43)	.34	44.4	0.11 (−0.44 to 0.67)	.69	7.3
**Baseline vitamin D status**								
Insufficient/deficient	11	924	−0.44 (−2.33 to 1.44)	.64	51.9	−0.08 (−0.83 to 0.99)	.86	31.3
Not clear^a^	16	2,886	−0.10 (−0.80 to 1.00)	.82	40.8	0.27 (−0.32 to 0.86)	.37	0
**Sample size**								
<200	22	2,240	−0.01 (−0.82 to 0.84)	.98	47.5	0.04 (−0.55 to 0.63)	.88	7.8
≥200	5	1,570	−0.03 (−1.41 to 1.35)	.96	13.5	0.46 (−0.35 to 1.28)	.27	0
**Type of vitamin D**								
Cholecalciferol	25	3,620	−0.01 (−0.76 to 0.73)	.98	46.2	0.25 (−0.24 to 0.74)	.32	7.3
Ergocalciferol	2	190	0.12 (−2.27 to 2.50)	.92	0	−0.73 (−2.63 to 1.17)	.45	0
**Frequency**								
Daily	18	2,053	−0.36 (−1.74 to 1.02)	.61	52.3	0.27 (−0.34 to 0.88)	.39	26.3
Weekly	3	416	0.91 (−0.99 to 2.81)	.35	0	−0.02 (−1.42 to 1.37)	.97	0
Fortnightly	1	71	3.69 (−0.49 to 7.87)	.08	—	1.54 (−1.81 to 4.89)	.37	—
Monthly	3	1,031	−1.02 (−2.71 to 0.67)	.24	0	−0.11 (−1.21 to 1.00)	.85	0
Single dose	2	239	1.30 (−1.84 to 4.43)	.42	0	0.25 (−1.72 to 2.21)	.80	0
**Duration^b^**								
<6 months	15	1,330	−0.23 (−1.71 to 1.26)	.76	55.8	0.11 (−0.58 to 0.80)	.75	28.7
≥6 months	10	2,241	−0.02 (−1.15 to 1.12)	.98	20.9	0.26 (−0.44 to 0.97)	.47	0.0
**Intervention type^c^**								
Vitamin D alone	18	2,774	0.16 (−0.69 to 1.00)	.72	0	0.25 (−0.30 to 0.80)	.38	6.0
Vitamin D + calcium	7	867	−0.65 (−3.66 to 2.37)	.68	70.4	−0.02 (−1.14 to 1.10)	.97	0
**Intervention amount^d^**								
≤800 IU/d	6	619	−1.91 (−4.24 to 0.42)	.15	57.9	−0.66 (−1.75 to 0.43)	.51	0
>800 IU/d	12	1,434	0.87 (−0.30 to 2.05)	.15	30.1	0.69 (−0.05 to 1.42)	.07	33.1
**Risk of bias**								
Low	12	1,564	−0.39 (−1.50 to 0.72)	.49	41.4	0.23 (−0.55 to 1.00)	.56	0
High	7	1,166	0.03 (−2.34 to 2.41)	.98	63.1	−0.38 (−1.34 to 0.58)	.44	18.2
Unclear	8	1,080	0.74 (−0.49 to 1.97)	.24	7.0	0.53 (−0.26 to 1.32)	.19	8.2

Abbreviations: —, not applicable/not calculated; CI, confidence interval; DBP, diastolic blood pressure; SBP: systolic blood pressure; WMD, weighted mean difference.

a “Not clear” is defined as articles that did not specify whether the subjects were overweight/obese or vitamin D insufficient/deficient.

b Total number of studies in the subgroup is not equal to 27, because 2 trials supplemented vitamin D by single dose (49,54).

c Total number of studies in the subgroup is not equal to 27, because 2 trials supplemented vitamin D with other mineral or multivitamin nutrient (44,45).

d This subgroup restricted to trials with daily administration.

## Discussion

This meta-analysis of cohort studies suggested an inverse association between 25(OH)D levels and incident hypertension, with hypertension risk reduced by 7% per 25 nmol/L increment in 25(OH)D levels. Meanwhile, summary data of RCTs indicated no evidence of blood pressure reduction by supplementation with vitamin D, a finding consistent with subgroup analyses based on baseline overweight/obese status, baseline 25(OH)D level, follow-up duration, and intervention dose.

The findings from numerous observational studies have shown that sufficient vitamin D status is a protective factor for hypertension. Analysis of Mendelian randomization also provided the causal evidence for the effect of increased circulating 25(OH)D levels on reduced blood pressure levels and risk of hypertension (55). However, our subgroup analyses of the cohort studies produced inconsistent results, which indicated that the quantitative data failed to provide convincing evidence of the protective effect of vitamin D on hypertension. Meanwhile, most of the interventional studies did not provide consistent evidence of blood pressure benefit from supplementing with vitamin D (11–13,21,49,50,53). Given these findings, we speculate that the beneficial effect observed in cohort studies may be partly explained by the tendency that sufficient vitamin D levels are closely related to healthy lifestyle or study participants being young. It may be also in part because of the hypothesis that low 25(OH)D levels could be the result of sub-health status rather than a precursor of diseases. Furthermore, differences exist among the various methods used (ie, liquid chromatography-mass spectrometry; high-performance liquid chromatography; and enzymoimmunoassay, radioimmunoassay, and chemiluminescence immunoassays) and in the laboratories that measured 25(OH)D levels, which would also influence the accuracy of the study results (56).

Similar with our results, previous meta-analyses also showed no overall lowering effect of vitamin D supplementation on blood pressure (14–16,57). However, they suggested that vitamin D may show a beneficial effect on blood pressure in specific subgroups, such as older people, people whose dosage of vitamin D was high (>800 IU/d), short-term interventions (<6 months), or individuals with pre-existing cardiometabolic disease (14,16). A possible reason for this discrepancy is that the recruited populations of included studies had high heterogeneity. Therefore, we restricted this meta-analysis to analyses of apparently healthy individuals. We excluded trials that have targeted patients with hypertension, diabetes, cardiovascular disease, or other diseases, because the known or unknown interaction between vitamin D and antihypertensive or cardiovascular medications may mask or attenuate the small effects of blood pressure reduction.

Complicated factors such as baseline vitamin D status, intervention design, or adiposity may modify or blunt the beneficial effect on blood pressure of improving vitamin D levels. An increasing body of evidence supports the presence of thresholds in vitamin D status (58). Similarly, the approximately L-shaped relationship between 25(OH)D levels and hypertension risk in our meta-analysis showed that hypertension risk increased substantially below 75 nmol/L but remained marginally significant above 75 nmol/L, which suggests that subjects with vitamin D insufficiency or deficiency show higher response to supplementation. In addition, evidence showed a therapeutic effect of cholecalciferol only in vitamin D–depleted participants by decreasing their 24-hour blood pressure by 3–4 mm Hg (59). Therefore, we speculated that the protective effect would only appear in subjects with low vitamin D levels. Indeed, we classified the studies according to their baseline vitamin D status, but the results indicated that vitamin D supplementation had no apparent effect on blood pressure, regardless of its baseline status. This finding is in accord with a recent meta-analysis that used individual patient data (15). However, considering that the number of people with low vitamin D levels may be insufficient in our study, further trials are needed to verify this finding.

Individuals who are taking vitamin D supplements should do so for at least 6 months to reach the maximum attained 25(OH)D level (60). It is reasonable to assume that the effect of vitamin D is time-dependent. However, our findings from subgroup analyses of RCTs suggested that response of blood pressure to vitamin D is independent of interventional duration (<6 months and ≥6 months). Similar findings have been reported (16,61). Considering these findings, we still cannot rule out that the duration of vitamin D intervention is insufficient to detect any slight but significant reduction in blood pressures, especially in the apparently healthy subjects whose normal values are less likely to be further improved. It is worth noting that until June 2019 only one RCT lasting up to 2 years was included in our study; therefore, a protective effect of longer intervention could not be studied adequately. Future RCTs with longer follow‐up duration are needed to provide in-depth insight into the long‐term benefits of vitamin D supplementation.

The optimal dose for vitamin D supplementation would influence the effect on blood pressure. A 4-arm trial conducted in African Americans reported dose-dependent reductions in SBP after 3 months of cholecalciferol supplementation with 1,000 IU, 2,000 IU, and 4,000 IU per day (0.66 mm Hg, 3.4 mm Hg, and 4.0 mm Hg, respectively) (34). In addition, a meta-analysis synthesizing the results of 30 RCTs suggested that vitamin D supplementation at a dose of >800 IU/d reduced blood pressures significantly (14). Contrary to these results, we did not find the dose–response relationship for vitamin D on blood pressure. We should consider the possibility that the supplementary doses in most included trials may be larger or smaller to observe a beneficial effect. Further studies are needed to explore the potential quantitative model.

This meta-analysis of RCTs included 3,810 people from the general population, which provides a substantial statistical power to detect the potential effects and thereby enhances the generalizability of our findings. However, our study also contains several potential limitations. First, because most studies did not record the changes of diet, sun exposure or latitudes, genetic factors, and educational status, we are not able to answer the questions of whether these factors would modify the effect of the intervention. Second, there are several trials that did not reach enough power (they were below 80%) to detect any weak difference between interventional and placebo groups because of the small sample size and high rate of noncompliance (13,20,53). In addition, although we stratified the duration of follow-up (the maximum is 2.0 years) and found no significant difference between subgroups, it remains unclear whether there are any long-term (>2 years) effects of vitamin D to improve blood pressure levels. However, we may conclude that vitamin D supplementation will not affect blood pressure short-term.

The results of this meta-analysis indicate that supplementation with vitamin D does not lower blood pressure in the general population. On the basis of this finding, we do not recommend using vitamin D supplementation to prevent hypertension. However, future RCTs with long-term interventions and sufficient sample sizes of people with low vitamin D levels are needed to replicate this finding.

## References

[1] Holick MF . Vitamin D deficiency. N Engl J Med 2007;357(3):266–81. 10.1056/NEJMra070553 17634462

[2] Institute of Medicine Food and Nutrition Board. Dietary reference intakes for calcium and vitamin D. Washington (DC): The National Academies Press; 2011. 21796828

[3] Jayedi A , Rashidy-Pour A , Shab-Bidar S . Vitamin D status and risk of dementia and Alzheimer’s disease: a meta-analysis of dose-response. Nutr Neurosci 2019;22(11):750–9. 29447107 10.1080/1028415X.2018.1436639

[4] Zhang R , Li B , Gao X , Tian R , Pan Y , Jiang Y , Serum 25-hydroxyvitamin D and the risk of cardiovascular disease: dose-response meta-analysis of prospective studies. Am J Clin Nutr 2017;105(4):810–9. 10.3945/ajcn.116.140392 28251933

[5] Ekmekcioglu C , Haluza D , Kundi M . 25-Hydroxyvitamin D status and risk for colorectal cancer and type 2 diabetes mellitus: a systematic review and meta-analysis of epidemiological studies. Int J Environ Res Public Health 2017;14(2):E127. 10.3390/ijerph14020127 28134804 PMC5334681

[6] Qi D , Nie XL , Wu S , Cai J . Vitamin D and hypertension: Prospective study and meta-analysis. PLoS One 2017;12(3):e0174298. 10.1371/journal.pone.0174298 28358827 PMC5373576

[7] Forman JP , Giovannucci E , Holmes MD , Bischoff-Ferrari HA , Tworoger SS , Willett WC , Plasma 25-hydroxyvitamin D levels and risk of incident hypertension. Hypertension 2007;49(5):1063–9. 10.1161/HYPERTENSIONAHA.107.087288 17372031

[8] Wang L , Ma J , Manson JE , Buring JE , Gaziano JM , Sesso HD . A prospective study of plasma vitamin D metabolites, vitamin D receptor gene polymorphisms, and risk of hypertension in men. Eur J Nutr 2013;52(7):1771–9. 10.1007/s00394-012-0480-8 23262750 PMC3609910

[9] van Ballegooijen AJ , Gansevoort RT , Lambers-Heerspink HJ , de Zeeuw D , Visser M , Brouwer IA , Plasma 1,25-dihydroxyvitamin D and the risk of developing hypertension: the Prevention of Renal and Vascular End-Stage Disease Study. Hypertension 2015;66(3):563–70. 10.1161/HYPERTENSIONAHA.115.05837 26195480

[10] Tomson J , Hin H , Emberson J , Kurien R , Lay M , Cox J , Effects of vitamin D on blood pressure, arterial stiffness, and cardiac function in older people after 1 year: BEST–D (Biochemical Efficacy and Safety Trial of Vitamin D). J Am Heart Assoc 2017;6(10):e005707. 10.1161/JAHA.117.005707 29066437 PMC5721827

[11] Scragg R , Slow S , Stewart AW , Jennings LC , Chambers ST , Priest PC , Long-term high-dose vitamin D3 supplementation and blood pressure in healthy adults: a randomized controlled trial. Hypertension 2014;64(4):725–30. 10.1161/HYPERTENSIONAHA.114.03466 24980662

[12] McMullan CJ , Borgi L , Curhan GC , Fisher N , Forman JP . The effect of vitamin D on renin–angiotensin system activation and blood pressure: a randomized control trial. J Hypertens 2017;35(4):822–9. 10.1097/HJH.0000000000001220 28033130 PMC5893307

[13] Daly RM , Nowson CA . Long-term effect of calcium-vitamin D(3) fortified milk on blood pressure and serum lipid concentrations in healthy older men. Eur J Clin Nutr 2009;63(8):993–1000. 10.1038/ejcn.2008.79 19156159

[14] Golzarand M , Shab-Bidar S , Koochakpoor G , Speakman JR , Djafarian K . Effect of vitamin D3 supplementation on blood pressure in adults: an updated meta-analysis. Nutr Metab Cardiovasc Dis 2016;26(8):663–73. 10.1016/j.numecd.2016.04.011 27287826

[15] Beveridge LA , Struthers AD , Khan F , Jorde R , Scragg R , Macdonald HM , ; D-PRESSURE Collaboration. Effect of vitamin D supplementation on blood pressure: a systematic review and meta-analysis incorporating individual patient data. JAMA Intern Med 2015;175(5):745–54. 10.1001/jamainternmed.2015.0237 25775274 PMC5966296

[16] Kunutsor SK , Burgess S , Munroe PB , Khan H . Vitamin D and high blood pressure: causal association or epiphenomenon? Eur J Epidemiol 2014;29(1):1–14. 10.1007/s10654-013-9874-z 24374742

[17] Kunutsor SK , Apekey TA , Steur M . Vitamin D and risk of future hypertension: meta-analysis of 283,537 participants. Eur J Epidemiol 2013;28(3):205–21. 10.1007/s10654-013-9790-2 23456138

[18] Bislev LS , Langagergaard Rødbro L , Bech JN , Pedersen EB , Kjaergaard AD , Ladefoged SA , The effect of vitamin D3 supplementation on markers of cardiovascular health in hyperparathyroid, vitamin D insufficient women: a randomized placebo-controlled trial. Endocrine 2018;62(1):182–94. 10.1007/s12020-018-1659-4 30043092

[19] Ramly M , Ming MF , Chinna K , Suboh S , Pendek R . Effect of vitamin D supplementation on cardiometabolic risks and health-related quality of life among urban premenopausal women in a tropical country—a randomized controlled trial. PLoS One 2014;9(10):e110476. 10.1371/journal.pone.0110476 25350669 PMC4211685

[20] Mitchell DM , Leder BZ , Cagliero E , Mendoza N , Henao MP , Hayden DL , Insulin secretion and sensitivity in healthy adults with low vitamin D are not affected by high-dose ergocalciferol administration: a randomized controlled trial. Am J Clin Nutr 2015;102(2):385–92. 10.3945/ajcn.115.111682 26156733 PMC4515870

[21] Bressendorff I , Brandi L , Schou M , Nygaard B , Frandsen NE , Rasmussen K , The effect of high dose cholecalciferol on arterial stiffness and peripheral and central blood pressure in healthy humans: a randomized controlled trial. PLoS One 2016;11(8):e0160905. 10.1371/journal.pone.0160905 27509187 PMC4980002

[22] Moghassemi S , Marjani A . The effect of short-term vitamin D supplementation on lipid profile and blood pressure in post-menopausal women: A randomized controlled trial. Iran J Nurs Midwifery Res 2014;19(5):517–21. 25400681 PMC4223970

[23] Seibert E , Lehmann U , Riedel A , Ulrich C , Hirche F , Brandsch C , Vitamin D_3_ supplementation does not modify cardiovascular risk profile of adults with inadequate vitamin D status. Eur J Nutr 2017;56(2):621–34. 10.1007/s00394-015-1106-8 26621634

[24] Sluyter JD , Camargo CA Jr , Stewart AW , Waayer D , Lawes CMM , Toop L , Effect of monthly, high-dose, long-term vitamin d supplementation on central blood pressure parameters: a randomized controlled trial substudy. J Am Heart Assoc 2017;6(10):e006802. 10.1161/JAHA.117.006802 29066444 PMC5721873

[25] Hutton B , Salanti G , Caldwell DM , Chaimani A , Schmid CH , Cameron C , The PRISMA extension statement for reporting of systematic reviews incorporating network meta-analyses of health care interventions: checklist and explanations. Ann Intern Med 2015;162(11):777–84. 10.7326/M14-2385 26030634

[26] Hamling J , Lee P , Weitkunat R , Ambühl M . Facilitating meta-analyses by deriving relative effect and precision estimates for alternative comparisons from a set of estimates presented by exposure level or disease category. Stat Med 2008;27(7):954–70. 10.1002/sim.3013 17676579

[27] Hartemink N , Boshuizen HC , Nagelkerke NJ , Jacobs MA , van Houwelingen HC . Combining risk estimates from observational studies with different exposure cutpoints: a meta-analysis on body mass index and diabetes type 2. Am J Epidemiol 2006;163(11):1042–52. 10.1093/aje/kwj141 16611666

[28] Wells GA , O’Connell D , Peterson J , Welch V , Losos M , Tugwell P . The Newcastle-Ottawa Scale (NOS) for assessing the quality of nonrandomised studies in meta-analyses. The Ottowa Hospital; 2017. http://www.ohri.ca/programs/clinical_epidemiology/oxford.asp. Accessed November 2, 2019.

[29] Cochrane handbook for systematic reviews of interventions, version 5.1.0 [updated March 2011]. The Cochrane Collaboration; 2011. http://www.handbook.cochrane.org. Accessed November 12, 2019.

[30] Egger M , Smith GD , Schneider M , Minder C . Bias in meta-analysis detected by a simple, graphical test. BMJ 1997;315(7109):629–34. 10.1136/bmj.315.7109.629 9310563 PMC2127453

[31] Orsini N , Bellocco R , Greenland S . Generalized least squares for trend estimation of summarized dose-response data. Stata J 2006;6(1):40–57. 10.1177/1536867X0600600103

[32] DerSimonian R , Laird N . Meta-analysis in clinical trials. Control Clin Trials 1986;7(3):177–88. 10.1016/0197-2456(86)90046-2 3802833

[33] Major GC , Alarie F , Doré J , Phouttama S , Tremblay A . Supplementation with calcium + vitamin D enhances the beneficial effect of weight loss on plasma lipid and lipoprotein concentrations. Am J Clin Nutr 2007;85(1):54–9. 17209177 10.1093/ajcn/85.1.54

[34] Forman JP , Scott JB , Ng K , Drake BF , Suarez EG , Hayden DL , Effect of vitamin D supplementation on blood pressure in blacks. Hypertension 2013;61(4):779–85. 10.1161/HYPERTENSIONAHA.111.00659 23487599 PMC3775458

[35] Skaaby T , Husemoen LL , Pisinger C , Jørgensen T , Thuesen BH , Fenger M , Vitamin D status and changes in cardiovascular risk factors: a prospective study of a general population. Cardiology 2012;123(1):62–70. 10.1159/000341277 22986625

[36] van Ballegooijen AJ , Kestenbaum B , Sachs MC , de Boer IH , Siscovick DS , Hoofnagle AN , Association of 25-hydroxyvitamin D and parathyroid hormone with incident hypertension: MESA (Multi-Ethnic Study of Atherosclerosis). J Am Coll Cardiol 2014;63(12):1214–22. 10.1016/j.jacc.2014.01.012 24480627 PMC3999436

[37] Margolis KL , Martin LW , Ray RM , Kerby TJ , Allison MA , Curb JD , ; Women’s Health Initiative Investigators. A prospective study of serum 25-hydroxyvitamin D levels, blood pressure, and incident hypertension in postmenopausal women. Am J Epidemiol 2012;175(1):22–32. 10.1093/aje/kwr274 22127681 PMC3291161

[38] Ke L , Graubard BI , Albanes D , Fraser DR , Weinstein SJ , Virtamo J , Hypertension, pulse, and other cardiovascular risk factors and vitamin D status in Finnish men. Am J Hypertens 2013;26(8):951–6. 10.1093/ajh/hpt051 23598420 PMC3816321

[39] Jorde R , Figenschau Y , Emaus N , Hutchinson M , Grimnes G . Serum 25-hydroxyvitamin D levels are strongly related to systolic blood pressure but do not predict future hypertension. Hypertension 2010;55(3):792–8. 10.1161/HYPERTENSIONAHA.109.143990 20065152

[40] Anderson JL , May HT , Horne BD , Bair TL , Hall NL , Carlquist JF , ; Intermountain Heart Collaborative (IHC) Study Group. Relation of vitamin D deficiency to cardiovascular risk factors, disease status, and incident events in a general healthcare population. Am J Cardiol 2010;106(7):963–8. 10.1016/j.amjcard.2010.05.027 20854958

[41] Wamberg L , Kampmann U , Stødkilde-Jørgensen H , Rejnmark L , Pedersen SB , Richelsen B . Effects of vitamin D supplementation on body fat accumulation, inflammation, and metabolic risk factors in obese adults with low vitamin D levels - results from a randomized trial. Eur J Intern Med 2013;24(7):644–9. 10.1016/j.ejim.2013.03.005 23566943

[42] Gagnon C , Daly RM , Carpentier A , Lu ZX , Shore-Lorenti C , Sikaris K , Effects of combined calcium and vitamin D supplementation on insulin secretion, insulin sensitivity and β-cell function in multi-ethnic vitamin D-deficient adults at risk for type 2 diabetes: a pilot randomized, placebo-controlled trial. PLoS One 2014;9(10):e109607. 10.1371/journal.pone.0109607 25299668 PMC4192133

[43] Zhu W , Cai D , Wang Y , Lin N , Hu Q , Qi Y , Calcium plus vitamin D3 supplementation facilitated fat loss in overweight and obese college students with very-low calcium consumption: a randomized controlled trial. Nutr J 2013;12(1):8. 10.1186/1475-2891-12-8 23297844 PMC3599592

[44] Maki KC , Rubin MR , Wong LG , McManus JF , Jensen CD , Lawless A . Effects of vitamin D supplementation on 25-hydroxyvitamin D, high-density lipoprotein cholesterol, and other cardiovascular disease risk markers in subjects with elevated waist circumference. Int J Food Sci Nutr 2011;62(4):318–27. 10.3109/09637486.2010.536146 21250901

[45] Toxqui L , Blanco-Rojo R , Wright I , Pérez-Granados AM , Vaquero MP . Changes in blood pressure and lipid levels in young women consuming a vitamin D-fortified skimmed milk: a randomised controlled trial. Nutrients 2013;5(12):4966–77. 10.3390/nu5124966 24317556 PMC3875909

[46] Salehpour A , Shidfar F , Hosseinpanah F , Vafa M , Razaghi M , Hoshiarrad A , Vitamin D3 and the risk of CVD in overweight and obese women: a randomised controlled trial. Br J Nutr 2012;108(10):1866–73. 10.1017/S0007114512000098 22317756

[47] Zittermann A , Frisch S , Berthold HK , Götting C , Kuhn J , Kleesiek K , Vitamin D supplementation enhances the beneficial effects of weight loss on cardiovascular disease risk markers. Am J Clin Nutr 2009;89(5):1321–7. 10.3945/ajcn.2008.27004 19321573

[48] Wood AD , Secombes KR , Thies F , Aucott L , Black AJ , Mavroeidi A , Vitamin D3 supplementation has no effect on conventional cardiovascular risk factors: a parallel-group, double-blind, placebo-controlled RCT. J Clin Endocrinol Metab 2012;97(10):3557–68. 10.1210/jc.2012-2126 22865902

[49] Witham MD , Adams F , Kabir G , Kennedy G , Belch JJ , Khan F . Effect of short-term vitamin D supplementation on markers of vascular health in South Asian women living in the UK—a randomised controlled trial. Atherosclerosis 2013;230(2):293–9. 10.1016/j.atherosclerosis.2013.08.005 24075759

[50] Gepner AD , Ramamurthy R , Krueger DC , Korcarz CE , Binkley N , Stein JH . A prospective randomized controlled trial of the effects of vitamin D supplementation on cardiovascular disease risk. PLoS One 2012;7(5):e36617. 10.1371/journal.pone.0036617 22586483 PMC3346736

[51] Nagpal J , Pande JN , Bhartia A . A double-blind, randomized, placebo-controlled trial of the short-term effect of vitamin D3 supplementation on insulin sensitivity in apparently healthy, middle-aged, centrally obese men. Diabet Med 2009;26(1):19–27. 10.1111/j.1464-5491.2008.02636.x 19125756

[52] Pfeifer M , Begerow B , Minne HW , Nachtigall D , Hansen C . Effects of a short-term vitamin D(3) and calcium supplementation on blood pressure and parathyroid hormone levels in elderly women. J Clin Endocrinol Metab 2001;86(4):1633–7. 11297596 10.1210/jcem.86.4.7393

[53] Jorde R , Sneve M , Torjesen P , Figenschau Y . No improvement in cardiovascular risk factors in overweight and obese subjects after supplementation with vitamin D3 for 1 year. J Intern Med 2010;267(5):462–72. 10.1111/j.1365-2796.2009.02181.x 20141565

[54] Scragg R , Khaw KT , Murphy S . Effect of winter oral vitamin D3 supplementation on cardiovascular risk factors in elderly adults. Eur J Clin Nutr 1995;49(9):640–6. 7498100

[55] Vimaleswaran KS , Cavadino A , Berry DJ , Jorde R , Dieffenbach AK , Lu C , ; LifeLines Cohort Study investigators; International Consortium for Blood Pressure (ICBP); Cohorts for Heart and Aging Research in Genomic Epidemiology (CHARGE) consortium; Global Blood Pressure Genetics (Global BPGen) consortium; Caroline Hayward. Association of vitamin D status with arterial blood pressure and hypertension risk: a mendelian randomisation study. Lancet Diabetes Endocrinol 2014;2(9):719–29. 10.1016/S2213-8587(14)70113-5 24974252 PMC4582411

[56] Abu el Maaty MA , Hanafi RS , Aboul-Enein HY , Gad MZ . Design-of-experiment approach for HPLC analysis of 25-hydroxyvitamin D: a comparative assay with ELISA. J Chromatogr Sci 2015;53(1):66–72. 10.1093/chromsci/bmu017 24714142

[57] Pittas AG , Chung M , Trikalinos T , Mitri J , Brendel M , Patel K , Systematic review: vitamin D and cardiometabolic outcomes. Ann Intern Med 2010;152(5):307–14. 10.7326/0003-4819-152-5-201003020-00009 20194237 PMC3211092

[58] Scragg R . Emerging evidence of thresholds for beneficial effects from vitamin D supplementation. Nutrients 2018;10(5):E561. 10.3390/nu10050561 29751504 PMC5986441

[59] Larsen T , Mose FH , Bech JN , Hansen AB , Pedersen EB . Effect of cholecalciferol supplementation during winter months in patients with hypertension: a randomized, placebo-controlled trial. Am J Hypertens 2012;25(11):1215–22. 10.1038/ajh.2012.111 22854639

[60] Shab-Bidar S , Bours S , Geusens PP , Kessels AG , van den Bergh JP . Serum 25(OH)D response to vitamin D3 supplementation: a meta-regression analysis. Nutrition 2014;30(9):975–85. 10.1016/j.nut.2013.12.020 24993750

[61] Wu L , Sun D . Effects of calcium plus vitamin D supplementation on blood pressure: a systematic review and meta-analysis of randomized controlled trials. J Hum Hypertens 2017;31(9):547–54. 10.1038/jhh.2017.12 28230063

